# *Mycobacterium tuberculosis* Strains Are Differentially Recognized by TLRs with an Impact on the Immune Response

**DOI:** 10.1371/journal.pone.0067277

**Published:** 2013-06-26

**Authors:** Jenny Carmona, Andrea Cruz, Lucia Moreira-Teixeira, Carole Sousa, Jeremy Sousa, Nuno S. Osorio, Ana L. Saraiva, Stefan Svenson, Gunilla Kallenius, Jorge Pedrosa, Fernando Rodrigues, Antonio G. Castro, Margarida Saraiva

**Affiliations:** 1 Life and Health Sciences Research Institute (ICVS), School of Health Sciences, University of Minho, Braga, Portugal; 2 ICVS/3B’s - PT Government Associate Laboratory, Braga/Guimarães, Portugal; 3 Escola Universitária Vasco da Gama (EUVG), Coimbra, Portugal; 4 Department of Clinical Science and Education, Karolinska Institutet, Stockholm, Sweden; Emory University School of Medicine, United States of America

## Abstract

Tuberculosis associates with a wide spectrum of disease outcomes. The Beijing (Bj) lineage of *Mycobacterium tuberculosis* (Mtb) is suggested to be more virulent than other Mtb lineages and prone to elicit non-protective immune responses. However, highly heterogeneous immune responses were reported upon infection of innate immune cells with Bj strains or stimulation with their glycolipids. Using both *in vitro* and *in vivo* mouse models of infection, we here report that the molecular mechanism for this heterogeneity may be related to distinct TLR activations. Among this Mtb lineage, we found strains that preferentially activate TLR2, and others that also activate TLR4. Recognition of Mtb strains by TLR4 resulted in a distinct cytokine profile *in vitro* and *in vivo*, with specific production of type I IFN. We also uncover a novel protective role for TLR4 activation *in vivo*. Thus, our findings contribute to the knowledge of the molecular basis underlying how host innate immune cells handle different Mtb strains, in particular the intricate host-pathogen interaction with strains of the Mtb Bj lineage.

## Introduction

*Mycobacterium tuberculosis* (Mtb) causes tuberculosis (TB) and is responsible for about 2 million deaths annually [1]. One of the most intriguing aspects of Mtb infection is the spectrum of disease outcomes, ranging from several degrees of pathogenesis, latency or clearance [2], [3]. Various host factors, including malnutrition and co-infection with HIV, influence on the outcome of TB [2], [3]. Recently, the contribution of Mtb genetic variation to the heterogeneity of TB outcomes is gaining importance [4]. Although TB was thought to be caused by essentially identical bacteria, recent evidence showed that it consists of 6 major lineages, each associated with geographical human populations [5] and with highly variable types of inflammatory responses [6], [7]. Much interest has been recently devoted to the study of the heterogeneity of responses associated with the East Asian (Bejing/W) (Bj) lineage of Mtb [8]. Several studies associate Mtb Bj strains with more severe forms of TB, TB outbreaks, drug resistance and less efficacious BCG vaccination 8,9,10. Experimental infections with Mtb Bj strains show the induction of non-uniform immune responses [11], [12], [13], [14], [15], but the molecular bases underlying this variability remain largely unclear.

The initiation of the immune response to Mtb relies on bacterial sensing by pattern recognition receptors (PRRs), as TLRs, expressed by multiple cell types including macrophages and dendritic cells (DCs) [16], [17]. Recognition of Mtb by TLRs triggers various intracellular signaling cascades, ultimately inducing the production of cytokines, chemokines and antimicrobial molecules [18], [19]. Evidence demonstrating a role for TLR activation in the control of Mtb comes from studies showing a lethal infection in mice lacking the master TLR signaling adaptor MyD88 [20], [21], [22]. However, the contribution of individual TLRs awaits further clarification. TLR2 is central for *in vitro* activation of the innate immune response [16], and was shown to impact the maintenance of Th17 cells in the lung of Mtb-infected animals [23] and granuloma formation [23], [24], but not bacterial burden [23], [24], [25], [26]. The role of other TLRs, such as TLR4 and TLR9, has been less addressed [25], [27], [28], [29]. In humans, the association of TLR polymorphisms with susceptibility to TB remains to be confirmed/needs further studies [19]. Different polymorphisms in the human *TLR2* gene were reported to associate with increased susceptibility to TB [30], [31], [32], [33], whereas such association was not observed in other studies [34], [35], [36], [37]. Furthermore, a MAL/TIRAP functional variant, affecting signaling through TLR2, was shown to be protective in TB [38]. Regarding TLR4, while genetic polymorphisms in an Asian Indian population were linked to an increased susceptibility and severity of pulmonary TB [39], in Gambian, Indian or Chinese TB patients no association was found [36], [37], [40]. This discrepancy might be explained on the basis of a dynamic host-pathogen genetic and pathogen phenotypic interplay, as postulated from studies involving *TLR2* polymorphisms [41].

Herein we show that some Mtb strains preferentially activate TLR2, whereas others activate TLR4. This differential recognition of Mtb strains impacts on both *in vitro* and *in vivo* immune responses, specifically inducing the production of different arrays of pro- and anti-inflammatory cytokines. Our findings present evidence that differential TLR recognition evoked by different Mtb strains may be decisive for shaping of innate immune responses *in vitro* and *in vivo* and possibly contributing for the outcome of Mtb infection.

## Materials and Methods

### Ethics Statement

All animal experiments were performed in strict accordance with the recommendations of the European Union Directive 86/609/EEC. This study was previously approved by the Portuguese National Authority for Animal Health - *Direcção Geral de Veterinária* (ID: DGV 594 from 1^st^ June 2010). Mice were humanely euthanized by cervical dislocation and every effort was made to minimize suffering.

### Animals

Eight to 12-week-old female C57BL/6 (BL6) mice, obtained from Charles River (Barcelona, Spain), Rag2^−/−^ from IGC, Portugal and TLR2^−/−^ or TLR4^−/−^ mice, bred at ICVS, were used.

### Bacteria

H37Rv Pasteur, HN878 and 03-265 were kind gifts from P.J. Cardona (*Unitat de Tuberculosi Experimental*, Barcelona), A. O’Garra (MRC-NIMR, UK) and F. Portaels (Institute of Tropical Medicine, Belgium), respectively. All Mtb strains were handed in the same way. Mtb strains were grown in 7H9 liquid media for 7–10 days and then diluted into Proskauer Beck medium with 0.05% Tween 80 for further expansion. When in mid-log phase bacterial stocks were frozen in 1 ml aliquots at –80°C. A few days later, aliquots of Mtb stocks were thawed and used to control for bacterial load. For this, serial dilutions of 6 frozen vials of each strain were plated in 7H11 agar plates and viable bacteria (colony forming units) counted after 3 weeks of incubation at 37°C. The obtained value was used to calculate the concentration of each strain stock used. All stocks were checked for Endotoxin contamination using the limulus assay (Sigma) and found to be negative. To infect macrophages or animals the frozen Mtb stocks were thawed and directly diluted in cDMEM (in vitro studies) or PBS (in vivo infections), considering the obtained value for CFU to calculate the volume of each strain stock needed for a specific moi.

### Genetic Analysis

Genomic DNA from each strain was subjected to PCR amplification as previously described [42] to determine whether the genomic deletions (RD105, RD181, RD150 and RD142) were present or absent.**.**

### Culture of BMDM

Bone marrow cells from WT, TLR2^−/−^ or TLR4^−/−^ animals were isolated from femurs and tibia and differentiated in the present of LCCM as previously described [43]. On day 7, cells were collected and infected with Mtb strains at a multiplicity of infection (moi) of 2 or with increasing moi (from 0.5 to 5) when indicated. At specific time-points post-infection, total RNA was isolated or supernatants collected and cytokine expression assessed by real-time PCR or ELISA.

### Quantitative Real Time-PCR analysis

Total RNA from infected lungs or cultured BMDMs was extracted with TRIzol® Reagent (Invitrogen) according to the manufacturer’s instructions. cDNA was synthesized and analyzed by real-time PCR as described previously [23]. Target gene mRNA expression was quantified using SYBR green (Qiagen) and specific oligonucleotides for each cytokine and normalized to the ubiquitin mRNA levels [23].

### ELISA

Supernatants from Mtb-infected BMDM cultures were screened for TNF, IL-6, IL-12p40, IL-10, IL-1β or IFN-β using commercially available kits.

### Experimental Infection

Mice were anesthetized with Ketamine/Medetomidine and infected intranasally with 100 cfu of each Mtb strain as reported previously [23]. All animal experiments were performed according to the European Union Directive 86/609/EEC and were previously approved by the National Authority *Direcção Geral de Veterinária*.

### Bacterial Load Determination

At specific time points post-infection, mice were sacrificed and the organs aseptically excised and homogenized in PBS. Serial dilutions of the organ homogenate were plated on nutrient 7H11 agar. Bacterial cfu were counted after 3 weeks of incubation at 37°C.

### iNOS Detection by Immunofluorescence

Caudal lobes of lungs from WT or TLR4^−/−^ infected mice were inflated with 3.8% phosphate-buffered formalin, fixed for 1 week and embedded in paraffin. Sections of 4µm thickness were used to detect iNOS by immunofluorescence with a goat anti-mouse antibody (M-19G, Santa Cruz) and detected with Alexa Fluor 568-conjugated polyclonal rabbit anti-goat (Invitrogen). DAPI (4′,6-diamino-2-phenylindole hydrochloride) was used to counterstain tissues and to detect nuclei.

### Statistics

Data are expressed as means ± SEM. Statistical significance was calculated by using two-tailed Student t-test. Values of p≤0.05 were considered statistically significant.

## Results

### *M. tuberculosis* Beijing Strains are Differentially Recognized by TLR2 or TLR4

**Figure 1:**
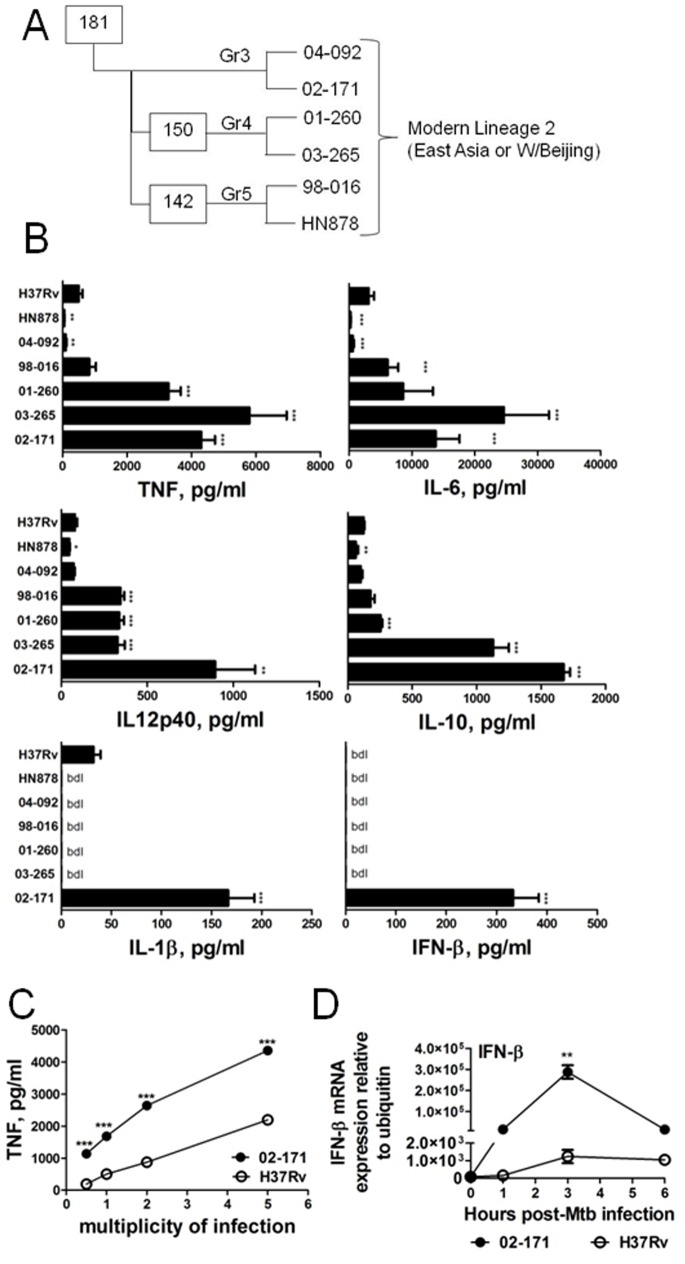
Mtb strains induce heterogeneous cytokine profiles in macrophages. (A) Phylogenetic distribution of the studied Mtb strains within the different subgroups of the Bj family defined by the presence or absence of the genomic deletions RD105, RD181, RD150, and RD142. (B) WT BMDM were infected with the laboratory reference strain (H37Rv) or with various Mtb strains of the Bj lineage (HN878 to 02-171). Six hours post-infection supernatants were harvested and the production of the indicated cytokines assessed by immunoassay. Each bar represents Mean±SEM of triplicate wells in three independent experiments. bdl, below detection level. (C) WT BMDM were infected with Mtb strains H37Rv (open circles) or 02-171 (close circles) at different moi. Six hours post-infection the supernatant of the infected cultures was harvested and the amount of secreted TNF measured by immunoassay. Each time point represents Mean±SEM of triplicate wells. (D) WT BMDM were infected with Mtb strains H37Rv (open circles) or 02-171 (close circles) with a moi of 2 and at different time points post-infection, RNA was isolated and the expression of IFN-β determined relatively to ubiquitin. Each time point represents Mean±SEM of triplicate wells. The statistics analysis, determined by the Student’s t-test, refers to differences between each strain and H37Rv (*,p≤0.05; **,p≤0.01; ***,p≤0.001). All bacterial stocks were handed in the same way, as detailed in Materials and Methods.

The Bj lineage of Mtb is known to induce heterogeneous immune responses [6], [7], [9], [11], [12], [13], [14], [15]. In the search for the host molecular basis underlying this heterogeneity, we started by comparing the induction of cytokine production by bone marrow-derived macrophages (BMDM) upon stimulation with a collection of phylogenetically diverse Mtb isolates of the Bj lineage (Figure 1A). Our analysis of cytokine production by infected cells was performed at 6 h post-infection, in order to focus on the events triggered by the innate immune recognition of Mtb strains, thus minimizing autocrine effects likely to occur at later times. Among this collection of Mtb isolates, we found strains, including HN878, that were lower inducers of both pro- and anti-inflammatory cytokine production than H37Rv (Figure 1B), which is in line with previous reports [44]. However, we also found strains that induced higher cytokine production in macrophages than H37Rv (Figure 1B). Overall the highest cytokine amounts were detected in macrophages infected with Mtb strain 02-171. This strain induced significantly higher TNF production than Mtb H37Rv at all moi tested (Figure 1C). The differences observed in the TNF production between Mtb strains H37Rv and 02-171 were not related to specific stocks, as independent stocks prepared by independent operators yielded the same results (Figure S1A). Furthermore, the differences in the cytokine responses induced in macrophages upon infection with different Mtb strains was not due to differential infection rate or bacterial uptake (Figure S1B).

Interestingly, Mtb strain 02-171 was unique in its ability to induce the secretion of IFN-β by macrophages (Figure 1B). To make sure that the differences observed were not due to delays in the kinetic profile of IFN-β production induced by Mtb strains H37Rv or 02-171, we measured IFN-β in the supernatants of 24 h infected macrophages and the same outcome was observed; however, at this time point, Mtb H37Rv induced a robust production of TNF, IL-10 and IL-1β by infected macrophages (data not shown). Furthermore, the kinetic analysis of IFN-β transcription upon infection of macrophages with Mtb strains, showed that strain 02-171 induced high levels of expression, whereas for the reference strain, H37Rv, only negligible transcriptional induction of this gene was observed (Figure 1D).

**Figure 2:**
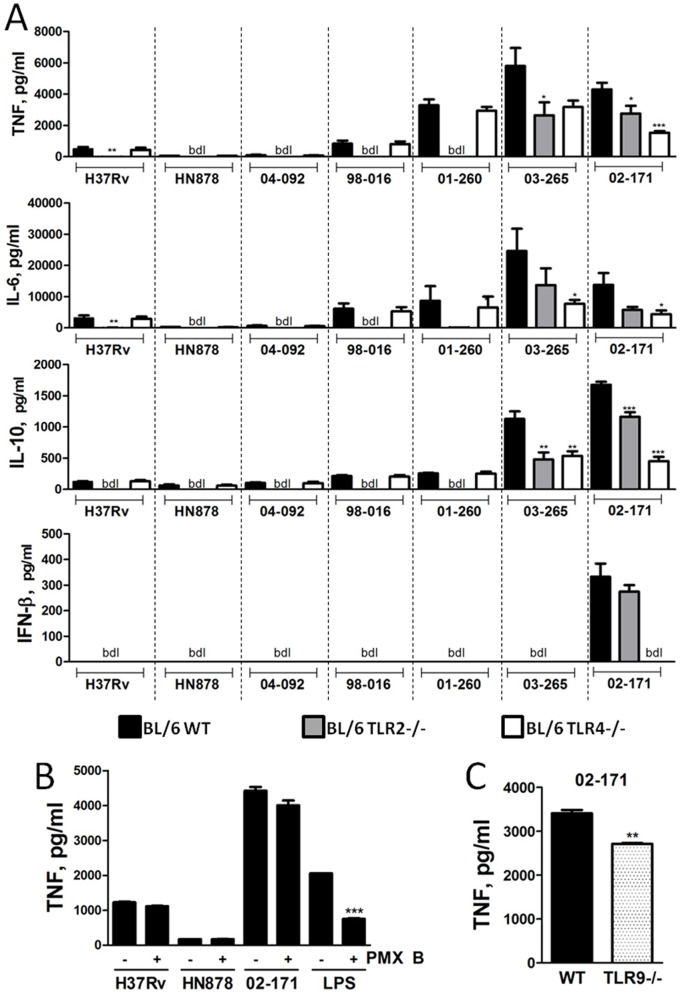
Mtb strains specifically activate TLR2 or TLR4 for cytokine induction. (A) BMDM generated from WT (black bars), TLR2^−/−^ (grey bars) or TLR4^−/−^ (white bars) mice were infected with various Mtb strains and cytokine secretion measured as in Figure 1B. Each bar represents Mean± SEM of triplicate wells in three independent experiments. bdl, below detection level. (B) WT BMDM were infected with Mtb strains H37Rv, HN878 or 02-171 (moi of 2) or stimulated with LPS (25 ng/ml) in the absence (-) or presence (+) of polymyxin B (PMX B, 5 µg/ml). Six hours post-infection the amount of TNF secreted by macrophages was determined by immunoassay. Each bar represents Mean±SEM of triplicate wells. (C) WT or TLR9^−/−^ BMDM were infected with Mtb strain 02-171 (moi of 2). Six hours post-infection the supernatant of the infected cultures was harvested and the amount of secreted TNF measured by immunoassay. Each bar represents Mean±SEM of triplicate wells. The statistics analysis, determined by the Student’s t-test, refers to differences between TLR2^−/−^ or TLR4^−/−^ and WT macrophages for each strain in (A), to differences between the absence or presence of polymyxin B in (B) and to differences between TLR9^−/−^ and WT macrophages in (C) (*,p≤0.05; **,p≤0.01; ***,p≤0.001).

**Figure 3:**
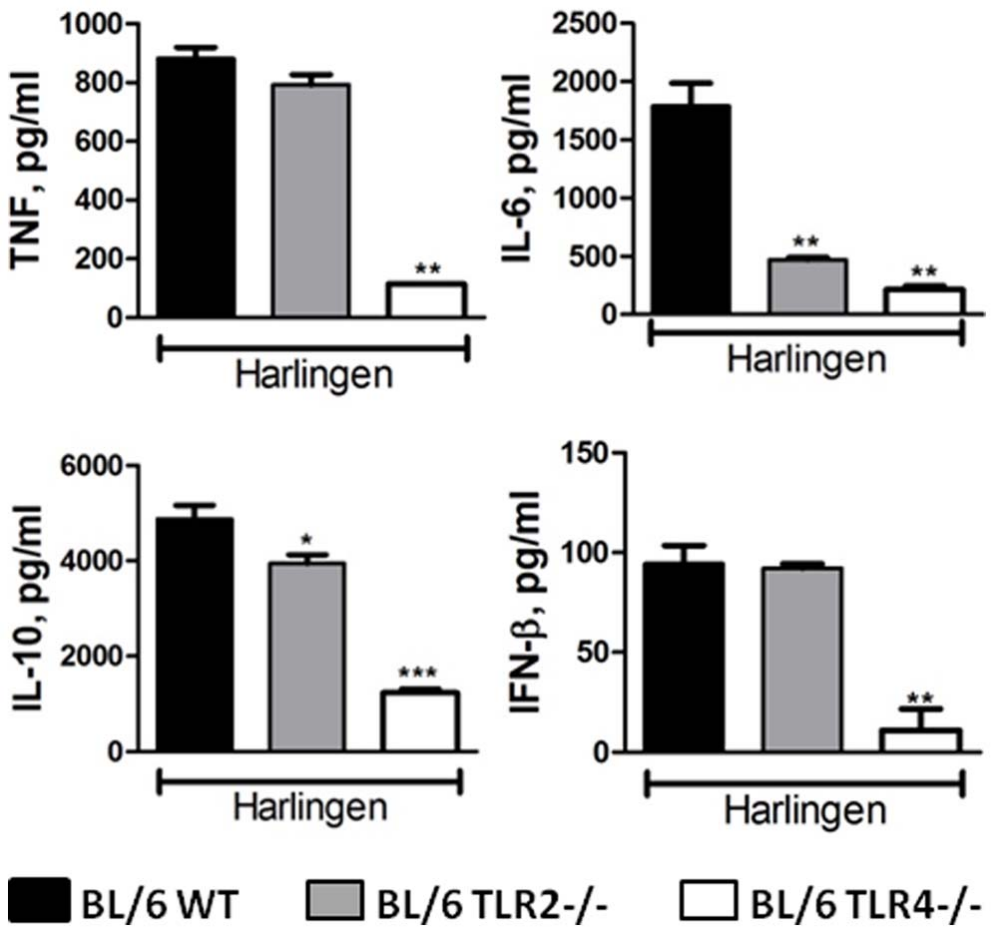
Mtb strain Harlingen preferentially activates macrophages via TLR4. BMDM generated from WT (black bars), TLR2^−/−^ (grey bars) or TLR4^−/−^ (white bars) mice were infected with Mtb strain Harlingen (moi of 2) and cytokine secretion measured as in Figure 1B. Each bar represents Mean±SEM of triplicate wells. The statistics analysis, determined by the Student’s t-test, refers to differences between TLR2^−/−^ or TLR4^−/−^ and WT macrophages (*,p≤0.05; **,p≤0.01; ***,p≤0.001). The data are representative of three independent experiments.

Considering the aforementioned unique secretion of IFN-β by macrophages infected with Mtb strain 02-171 and that TLRs are important in the recognition of Mtb [16], [19], we hypothesized that the observed heterogeneity might be due to differential activation of TLRs, particularly of TLR4, by Mtb isolates. To test this hypothesis, we infected BMDM of WT, TLR2^−/−^ and TLR4^−/−^ origin, with the panel of Mtb Bj strains under study. Interestingly, we found that the induction of cytokine production by macrophages was due, for most of the studied strains, to TLR2 activation, as expected from previous studies [19], whereas in the case of Mtb strain 02-171 it was mostly dependent on TLR4 activation (Figure 2A). In particular, the ability of Mtb strain 02-171 to induce the secretion of IFN-β by infected BMDM was totally dependent of TLR4 signals (Figure 2A, bottom panel). Kinetic mRNA analysis of WT BMDM infected with Mtb strains H37Rv or 02-171 revealed a pattern of variation consistent with the protein analysis (Figure S1C). The production of TNF by macrophages infected with Mtb strain 02-171 depended on the activation of TLR4 for all tested moi (Figure S1D). The presence of polymyxinB (an endotoxin inhibitor), at a concentration that inhibited over 50% of the TNF production by BMDM in response to LPS, did not interfere with the response of BMDM infected with Mtb strains H37Rv, HN878 or 02-171 (Figure 2B). In addition to TLR2 and TLR4, TLR9 has also been involved in Mtb recognition [19], [25]. We tested whether Mtb strain 02-171 varied in its ability to induce TLR9 signals and found that the contribution of this receptor to TNF induction was minimal (Figure 2C), in line with previous reports for Mtb strain H37Rv [25]. Recognition by TLR4 was not unique to the Mtb Bj lineage as an isolate of the Euro-American lineage (Mtb strain Harlingen) also triggered cytokine production in macrophages via TLR4, including the production of IFN-β (Figure 3).

Altogether, we show that distinct Mtb isolates of the Bj lineage are recognized by different TLRs in macrophages, resulting in the production of singular cytokine profiles, the most striking difference being the presence or absence of type I IFN. Thus, we propose the differential TLR recognition of Mtb strains as a possible molecular determinant of heterogeneous innate immune responses to Mtb.

### *In vivo* Analysis of Infection with *M. tuberculosis* TLR4-activating 02-171 Strain

**Figure 4:**
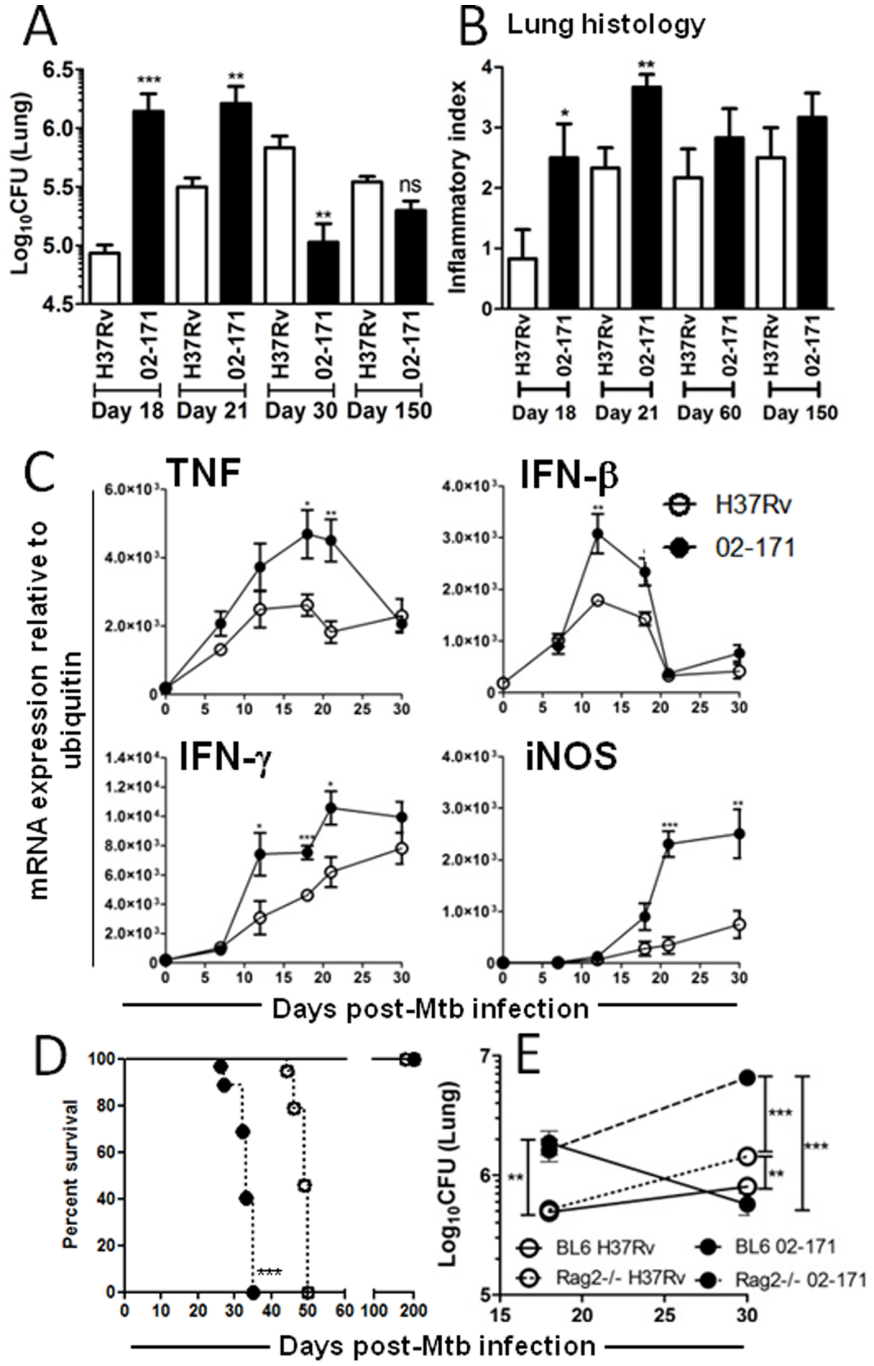
Mtb Bj strain 02-171 is more virulent during the innate immune response than H37Rv. (A) WT mice were intranasally infected with Mtb strains H37Rv (white bars) or 02-171 (black bars) and at the indicated time points post-infection, lung cell suspensions were prepared, diluted and plated into 7H11 agar to determine the number of mycobacterial CFUs in the lung. The bacterial burden 24 h post-infection was Log10(CFU) 2.67±0.57 and Log10(CFU) 2.45±0.76 (Mean±SEM for 6 animals per strain) for Mtb strains H37Rv or 02-171 infected mice, respectively. (B) WT mice were infected as before and, at the indicated time points post-infection, sections were prepared from formalin-fixed lungs. HE stained tissue was blindly analysed and lung inflammation scored for each animal within the group (6 animals). Inflammation scores were: absent = 0; mild = 1; abundant = 2; severe = 3; exacerbated = 4. (C) WT mice were infected with Mtb strains H37Rv (open circles) or 02-171 (close circles), RNA extracted from the lung tissue and the expression of TNF, IFN-β, IFN-γ and iNOS analyzed by quantitative real-time PCR and normalized to the expression of ubiquitin. Data represented for day 0 correspond to uninfected animals. For A, B and C data points show the Mean±SEM for 5 mice per group and the significance was determined by the Student’s t-test (*,p≤0.05; **,p≤0.01; ***,p≤0.001) for each time point, between Mtb strains 02-171 and H37Rv. The data are representative of three (A and B) or two (C) independent experiments. (D) Kaplan-Meier survival analysis of WT (solid lines) or Rag2^−/−^ (dashed lines) mice infected intranasally with Mtb strains H37Rv (open circles) or 02-171 (close circles). H37Rv-infected mice showed improved survival (49 days vs 33 days with 02-171-infected mice), as calculated by log-rank test (***,p≤0.0001). WT animals infected in parallel survived up to day 200 post-infection. The data are representative of two independent experiments. (E) WT or Rag2^−/−^ mice were infected with Mtb strains H37Rv or 02-171 and lung CFU numbers determined on days 18 and 30 post-infection as indicated before. Data points show the Mean±SEM for 6 mice per group. The statistics analysis between the different groups was determined by the Student’s t-test (**,p≤0.01; ***,p≤0.001).

In view of the striking differences observed between the responses of macrophages to Mtb strains H37Rv versus 02-171, we next investigated the progression of *in vivo* infections with these two strains. As compared to H37Rv, the bacterial burden in lungs of mice infected with Mtb strain 02-171 was higher during the initial phase of infection (up to day 21 post-infection) (Figure 4A). However, this pulmonary load was significantly reduced between days 21 and 30, remaining stable and becoming closer to Mtb strain H37Rv for the duration of the experiment (up to day 150 post-infection) (Figure 4A). The increased bacterial burden observed in the first three weeks of infection for the Mtb strain 02-171 was accompanied by enhanced lung pathology, measured by the extent of lung infiltrates (Figure 4B). However, and as observed for the progression of bacterial burden (Figure 4A), the lung pathology was similar to both strains at later time points (Figure 4B). In line with our *in vitro* data (Figure 1B), the expression of cytokines at the site of infection was higher in the case of Mtb strain 02-171 as compared to H37Rv (Figure 4C). Interestingly, the lung expression of iNOS during infection with Mtb strain 02-171 was also higher than with H37Rv (Figure 4C). Altogether our data suggest that Mtb strain 02-171 may be more virulent for mice during the innate immune phase of the infection, although the adaptive immune response is efficient in inducing bacteriostasis. To further investigate whether Mtb strain 02-171 is indeed more virulent for mice than H37Rv during innate immunity, we infected Rag2^−/−^ mice with either strain and followed the progression of infection. Rag2^−/−^ mice infected with Mtb strain 02-171 succumbed earlier than H37Rv-infected ones (Figure 4D) and showed higher bacterial burdens at day 30 post-infection (Figure 4E). Our data thus show that Mtb strain 02-171 is more virulent than H37Rv as evidenced by a faster bacterial replication at early stages of infection, earlier killing of immune compromised hosts and enhanced lung pathology. However, Mtb strain 02-171 does induce an adaptive immune response that manages to reduce lung bacterial loads at later stages of infection.

### TLR4 Deficiency Impacts the Outcome of *in vivo* Infection by a TLR4-activating *M. tuberculosis* Strain

**Figure 5:**
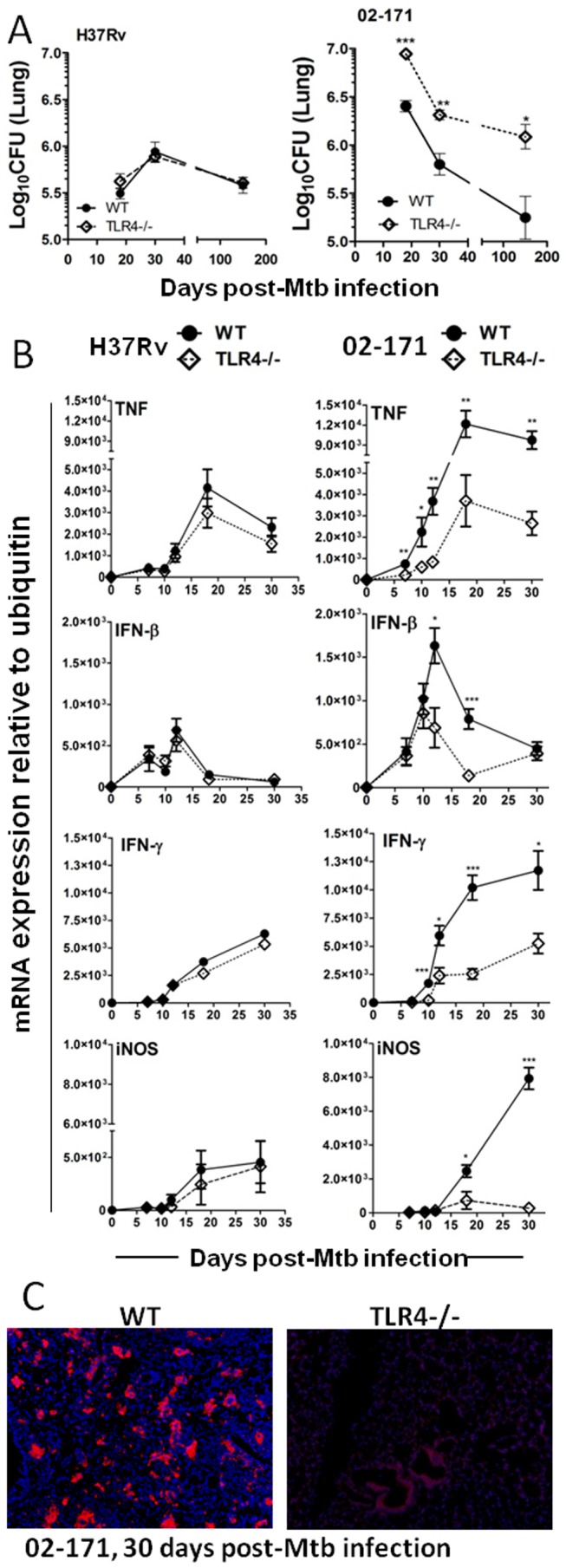
TLR4 deficiency compromises innate protection against Mtb infection by a TLR4-activating Mtb Bj strain. (A) WT (circles) or TLR4^−/−^ (diamonds) mice were infected intranasally with Mtb strains H37Rv (left panel) or 02-171 (right panel) and at the indicated time points the bacterial burden in the lungs determined as in Figure 4A. The bacterial burden 24 h post-infection was Log10(CFU) 2.25±0.68 and Log10(CFU) 2.10±0.28 for WT or TLR4^−/−^ infected with Mtb strain H37Rv (Mean±SEM for 6 animals per strain). As for Mtb strain 02-171 infected mice, the bacterial burden 24 h post-infection was Log10(CFU) 2.48±0.61 and Log10(CFU) 2.58±0.43 for WT or TLR4^−/−^ (Mean±SEM for 6 animals per strain), respectively. (B) WT (circles) or TLR4^−/−^ (diamonds) mice were infected with Mtb strains H37Rv (left panel) or 02-171 (right panel), and the expression of TNF, IFN-γ, IFN-β and iNOS analyzed by quantitative real-time PCR and normalized to the expression of ubiquitin. Data represented for day 0 correspond to uninfected animals. For A and B, data points show the Mean±SEM for six mice per group and the significance was determined by the Student’s t-test (*,p≤0.05; **,p≤0.01; ***,p≤0.001) for each time point and each Mtb strain between WT and TLR4^−/−^ mice. The data are representative of two independent experiments. (C) The production of iNOS in the lung tissue of WT or TLR4^−/−^ infected mice was determined for day 30 post-infection by immunofluorescence. Represented are 10× magnifications of one animal out of 5 per group.

We next sought to investigate whether the differences (Figure 4) observed in the immune response between Mtb strains H37Rv and 02-171 were related to the distinct activation of TLR4/TLR2 by the latter (Figure 2), by infecting BL6 WT or TLR4^−/−^ mice with either strain. Whilst absence of TLR4 did not impact the growth of Mtb strain H37Rv in mice, lack of this receptor led to a marked increase in the lung bacterial burden of Mtb strain 02-171-infected mice within the first two weeks post-infection (Figure 5A). After day 18 post-infection, when the acquired immune responses take control [45], [46], growth of Mtb strain 02-171 strain was controlled even in the absence of TLR4 (Figure 5A). However, the bacterial burdens remained higher in TLR4−/− than in WT mice and higher than the ones found in mice infected with H37Rv (Figure 5A). Of note, absence of TLR2 was dispensable for bacterial control during infections with either Mtb H37Rv or 02-171 strains (Figure S2A).

In line with a role for TLR4 in the *in vitro* recognition of Mtb strain 02-171 (Figure 2A), the absence of this TLR led to a marked reduction in the expression of several cytokines in infected lungs (Figure 5B). Indeed, in TLR4−/− infected lungs the level of cytokine expression induced by Mtb strain 02-171 was very much similar to that observed upon infection of WT mice with H37Rv (Figure 5B). Absence of TLR4 did not impact cytokine expression in infections with H37Rv (Figure 5B). Furthermore, absence of TLR4 also compromised iNOS gene expression (Figure 5B) and protein (Figure 5C) during the infection with Mtb strain 02-171. This finding may explain the inability of TLR4^−/−^ mice to control Mtb strain 02-171 infection to the same degree as BL6 WT mice (Figure 5A). In all, our *in vivo* data further support that TLR4 activation by certain Mtb strains contributes to cytokine induction in the lung of infected hosts and suggest a protective role for TLR4 limited to the innate immune response to a TLR4-activating strain of Mtb.

## Discussion

Infections by Mtb, which represent the second most common infectious cause of mortality and morbidity worldwide, are characterized by an intriguingly large spectrum of disease outcomes dictated by both host and pathogen genetic factors [2], [3]. Also fascinating is the marked heterogeneity of the immune response mounted by the host against Mtb strains of the 6 described lineages [2], [7], [44]. This topic is particularly relevant for Mtb strains of the Bj lineage, due to its possible association with disease severity [4], [8]. In this study, we provide evidence that differential innate recognition of Mtb strains stands as a relevant molecular mechanism associated with heterogeneous innate immune responses to different variants of this pathogen.

Several classes of PRRs are involved in the recognition of Mtb, including TLRs, C-type lectin receptors (CLRs), and Nod-like receptors (NLRs) [19]. Both MyD88 and CARD9 (the master adaptors of TLR and CLR signaling, respectively) are central for the development of protective immune responses to Mtb [20], [21], [22], [47]. Among the TLR family, TLR2, TLR4 and TLR9 are important molecules for the recognition of Mtb bacilli and their relevance during Mtb infection has been extensively studied. However, several authors have reported opposing results concerning the role of these receptors in Mtb infections, with mice lacking individual TLRs presenting either increased [24], [27], [29], [48] or unaltered [23], [26], [28] susceptibility to Mtb infection. To elucidate the possible joint action of these TLRs, studies were also performed with double and triple knock-out mice [25], [49]. Mice deficient for TLR2, 4 and 9 were shown to present a mild phenotype and control Mtb replication [25], [49], adding to the puzzling set of data describing TLR function during Mtb infection.

Comparing a panel of Mtb strains of the Bj lineage to the laboratory reference strain H37Rv, we demonstrate here that although most Mtb strains (including the reference strains H37Rv and HN878) were recognized by TLR2, Mtb strain 02-171 also activated macrophages via TLR4. Recognition of Mtb mediated by TLR4 was not associated with particular phylogeographical groups within the Bj lineage, as strains within the same group (eg. 04-092 and 02-171 strains from Gr3) preferentially activated different TLRs. However, such a scenario may be different if a more high-resolution tool for phylogenetic analysis [50] is used. TLR4 triggering was also not exclusive of the Bj lineage, as an Euro-American Mtb strain (Harlingen) was predominantly recognized by this TLR. Finally, the heterogeneity of the immune response and the differential TLR recognition reported were also not related to the profile of drug susceptibility of each strain [51].

TLR4 activation results in intracellular signals mediated by both MyD88 and TRIF, whereas TLR2 signals depend on MyD88 alone [18]. As a result of TRIF-pathway activation, a signaling complex involving IKK and TBK-1 is formed, which catalyze the phosphorylation of IRF3 and induce its nuclear translocation, leading to the transcription of *ifnβ* [18]. Consistent with this, secretion of IFN-β by Mtb-infected macrophages was only observed for TLR4-activating strains (02-171 and Harlingen). Taking into consideration a recent report showing that Mtb activates the DNA-dependent cytosolic surveillance pathway in macrophages, inducing signalling through Sting/TBK-1/IRF3 with consequent *ifnβ* transcription [52], our data now suggest that certain strains of Mtb are also able to enhance this *ifnβ* transcription via TLR4 activation. This finding may be relevant as type I IFN and IFN-inducible genes constitute a molecular signature associated with active pulmonary Mtb infection in humans [53]. Although the role of type I IFN during Mtb infection remains unclear [15], [54], [55], mice lacking type I IFN receptor present decreased bacterial burden and late mortality upon Mtb infection [15], [52], [54], [56]. Moreover, the virulence of certain Mtb strains was suggested to be correlated with their ability to induce type I IFN production *in vivo* [15], [54]. Our findings showing that Mtb strain 02-171 (a TLR4-activating and type I IFN inducer) was more virulent during the innate phase of the immune response than H37Rv in mice are in line with these observations. Nevertheless, in the absence of TLR4 we found a further increase on the lung bacterial burden of mice infected with Mtb strain 02-171, despite the lower induction of IFN-β expression. Also, Mtb strain Harlingen, another TLR4 activating strain, showed a degree of virulence comparable to H37Rv (Fig. S2B), pointing to the fact that the mechanisms underlying differences in virulence during the innate phase of the immune response are most likely multifactorial. On one hand, differences in virulence may result from the balance of cytokine responses induced by different strains. Indeed, Mtb strain 02-171 induced higher levels of both pro- and anti-inflammatory cytokines in the lung of infected animals, a balance that may favor susceptibility during the innate phase of the immune response. This balance was altered in the absence of TLR4, where we observed a decrease in the overall cytokine expression and in the expression and production of iNOS, accompanying an increase in bacterial burden. On the other hand, it is also possible that Mtb strain 02-171 shows a higher resistance to host innate killing mechanisms, thus escaping them and growing faster during the early phases of infection.

Our data suggest a possible dual role for TLR4 during infection with certain strains of Mtb, since its activation not only induced type I IFN, that may result in increased susceptibility [15], [52], [54], [56], but simultaneously induced the expression of protective cytokines (e.g. TNF) and of iNOS, a molecule known to be important for the control infection [57]. The participation of TLR4 appeared to predominate in the innate phases of the immune response. In contrast, the adaptive immunity was equally effective either in the presence or absence of TLR4, causing a significant reduction in CFU numbers observed after day 18 of infection. This finding is interesting, as previous reports on the role of TLR2 or TLR9 in Mtb infections evidenced a more pronounced effect in later stages of infection, in terms of granuloma formation [24], [27], [29], [48], Th17 cell maintenance [24], [27], [29], [48] or Th1 cell differentiation [24], [27], [29], [48]. Of note, in the absence of TLR4 we still observed cytokine expression in the lungs of mice infected with Mtb strain 02-171, suggesting that other PRRs, and/or that innate cells other than macrophages, harboring a different set of PRRs, must operate *in vivo*.

Despite the faster growth observed for Mtb strain 02-171 during the initial phase of the *in vivo* infection, at around day 30 we observed a sharp decrease on the lung bacterial load. This is an atypical observation for Mtb infections and the underlying cellular and molecular mechanisms are currently under investigation. It is possible that this strain of Mtb induces a T cell response with specific characteristics and dynamics that transiently lead to a better control of bacterial burden in the lung, thus allowing killing of the mycobacteria. In line with this hypothesis, our findings show that in a system where the acquired immune response fails, such as the Rag2^−/−^, Mtb strain 02-171 is more virulent and lethal than the reference strain H37Rv.

Our study brings forth a compelling amount of evidence showing that Mtb strains are recognized differently via TLR2 or TLR4 with possible implications on the *in vivo* innate immune responses to Mtb. It would be of the utmost interest to now pursue our findings in an effort to uncover the molecule(s) present in Mtb strain 02-171 that is/are able to activate TLR4. It will be also of interest to increase the number of strains tested in order to investigate how they cluster according to their ability to preferentially activate TLR2 vs TLR4. Our results may, at least in part, explain the discrepant data obtained by different groups addressing the association of TLR polymorphisms to TB susceptibility, highlighting the relevance of also investigating the relation between the infecting strain and the studied TLR polymorphism.

## Supporting Information

Figure S1 **Differential TLR recognition of Mtb strains determines the cytokine transcription profile of infected macrophages and is independent of the moi.** (A) WT BMDM were infected with two independent stocks of Mtb strains H37Rv (stocks 1 and 2) or 02-171 (stocks A and B) with a moi of 2. Six hours post-infection the supernatant of the infected BMDM was harvested and the amount of secreted TNF measured by immunoassay. Each bar represents Mean±SEM of triplicate wells. The statistics analysis was determined by the Student’s t-test (**,p≤0.01; ***,p≤0.001). (B) BMDM were infected with the indicated Mtb strains with a moi of 2, for 4 h. After this period of time, the wells were extensively washed, the cells lysed and the bacterial burden determined by CFU counting in 7H11 agar plates. Each bar represents Mean±SEM of six wells. (C) BMDM generated from WT (close circles), TLR2^−/−^ (open squares) or TLR4^−/−^ (open diamonds) mice were infected with Mtb strains H37Rv or 02-171 with a moi of 2 and at specific time points post-infection RNA was extracted and the expression of TNF, IL-6, IL-10 and IFN-β analyzed by quantitative real-time PCR and normalized to the expression of ubiquitin. Each time point represents Mean±SEM of triplicate wells. The statistics analysis, determined by the Student’s t-test, refers to differences between TLR2^−/−^ or TLR4^−/−^ and WT macrophages for each strain (*,p≤0.05; **,p≤0.01; ***,p≤0.001). (D) WT (close circles) or TLR4^−/−^ (open diamonds) BMDM were infected with Mtb strain 02-171 at different moi. Six hours post-infection the supernatant of the infected cultures was harvested and the amount of secreted TNF measured by immunoassay. Each moi analysed represents Mean±SEM of triplicate wells. The statistics analysis was determined by the Student’s t-test (***,p≤0.001).
(TIF)

Figure S2 **(A) Deficiency of TLR2 does not compromise the control of infection with Mtb strains H37Rv or 02-171.** WT (circles) or TLR2^−/−^ (squares) mice were infected intranasally with Mtb strains H37Rv or Bj 02-171 and at the indicated time points, lung cell suspensions were prepared, diluted and plated to determine the number of mycobacterial CFUs. Data points show the Mean±SEM for six mice per group. The values were found not to be significantly different as determined by the Student’s t-test. The data are representative of two independent experiments. **(B) Mtb strain 02-171 presents an increased in vivo growth as compared to H37Rv or Harlingen.** WT mice were infected intranasally with Mtb strains H37Rv (open circles), 02-171 (close circles) or Harlingen (triangles) and at the indicated time points, lung cell suspensions were prepared, diluted and plated to determine the number of mycobacterial CFUs. Data points show the Mean±SEM for six mice per group. The bacterial burden 24 h post-infection was Log10(CFU) 2.67±0.57, Log10(CFU) 2.45±0.76 and Log10(CFU) 2.51±0.46 (Mean±SEM for 6 (H37Rv or 02-171) or 5 (Harlingen) animals) for Mtb strains H37Rv, 02-171 or Harlingen infected mice, respectively. The statistics analysis was determined by the Student’s t-test (*,p<0.05; **p<0.01; ***,p≤0.001).
(TIF)
