# Supplementary figures and images for: Mycobacterium tuberculosis Strains Are Differentially Recognized by TLRs with an Impact on the Immune Response
Source: PLoS One. 2013 Jun 26;8(6):e67277. doi: 10.1371/journal.pone.0067277 (PMC3693941; doi:10.1371/journal.pone.0067277)

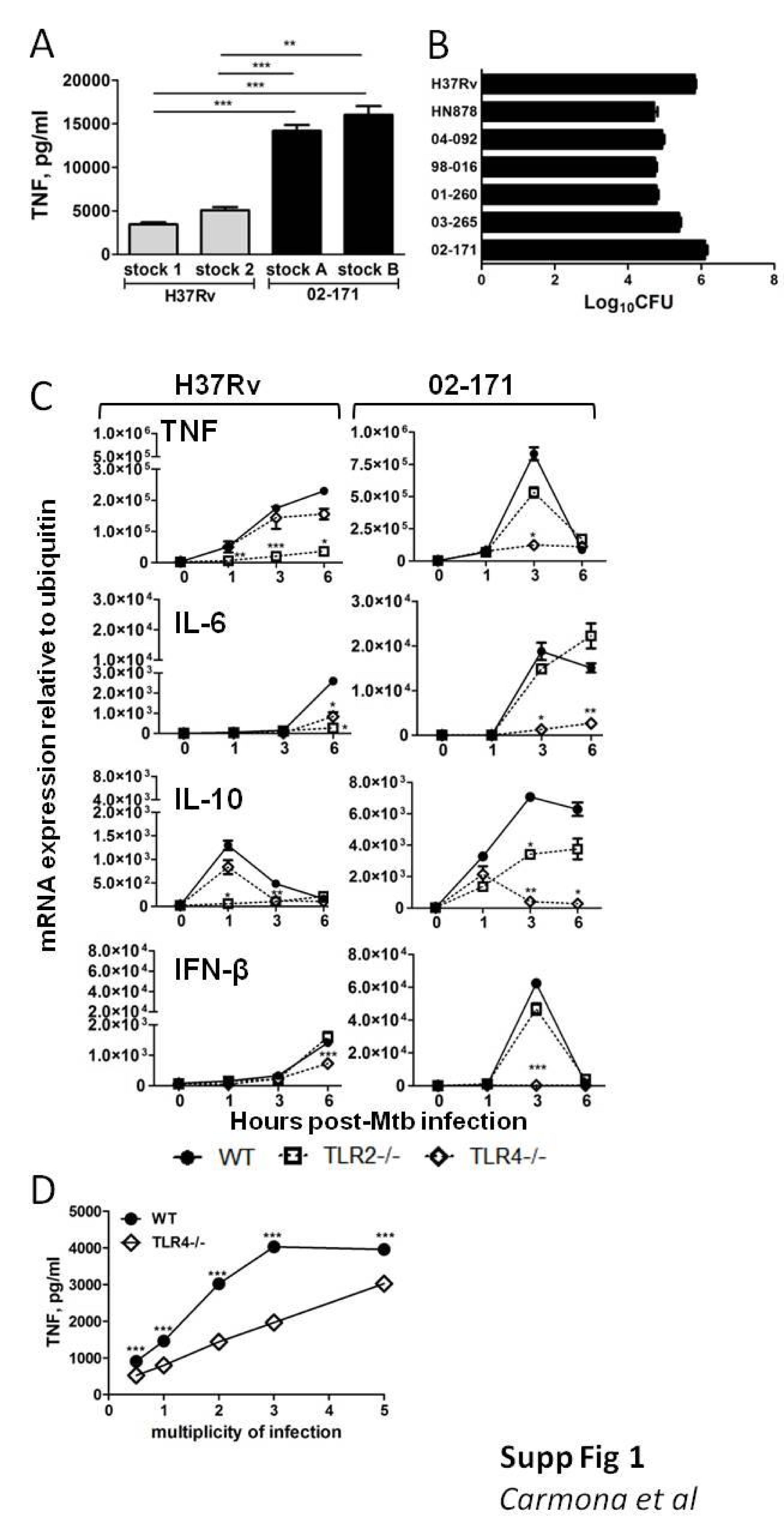

Supplement: Figure S1 — Differential TLR recognition of Mtb strains determines the cytokine transcription profile of infected macrophages and is independent of the moi. (A) WT BMDM were infected with two independent stocks of Mtb strains H37Rv (stocks 1 and 2) or 02-171 (stocks A and B) with a moi of 2. Six hours post-infection the supernatant of the infected BMDM was harvested and the amount of secreted TNF measured by immunoassay. Each bar represents Mean±SEM of triplicate wells. The statistics analysis was determined by the Student’s t-test (**,p≤0.01; ***,p≤0.001). (B) BMDM were infected with the indicated Mtb strains with a moi of 2, for 4 h. After this period of time, the wells were extensively washed, the cells lysed and the bacterial burden determined by CFU counting in 7H11 agar plates. Each bar represents Mean±SEM of six wells. (C) BMDM generated from WT (close circles), TLR2−/− (open squares) or TLR4−/− (open diamonds) mice were infected with Mtb strains H37Rv or 02-171 with a moi of 2 and at specific time points post-infection RNA was extracted and the expression of TNF, IL-6, IL-10 and IFN-β analyzed by quantitative real-time PCR and normalized to the expression of ubiquitin. Each time point represents Mean±SEM of triplicate wells. The statistics analysis, determined by the Student’s t-test, refers to differences between TLR2−/− or TLR4−/− and WT macrophages for each strain (*,p≤0.05; **,p≤0.01; ***,p≤0.001). (D) WT (close circles) or TLR4−/− (open diamonds) BMDM were infected with Mtb strain 02-171 at different moi. Six hours post-infection the supernatant of the infected cultures was harvested and the amount of secreted TNF measured by immunoassay. Each moi analysed represents Mean±SEM of triplicate wells. The statistics analysis was determined by the Student’s t-test (***,p≤0.001). (TIF) [file pone.0067277.s001.tif]

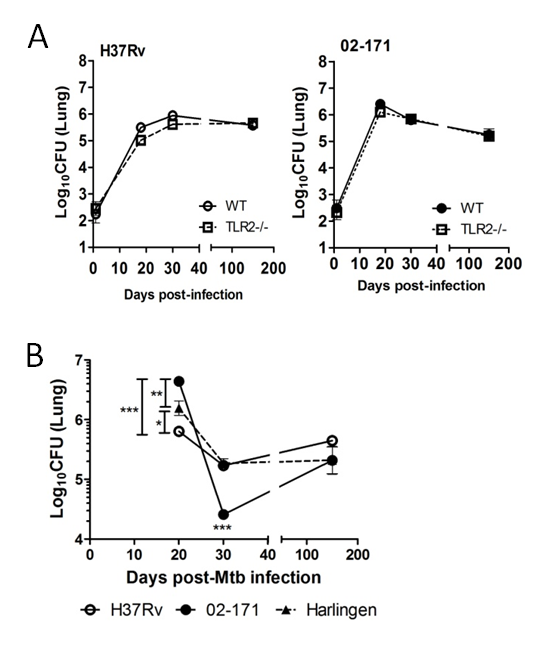

Supplement: Figure S2 — (A) Deficiency of TLR2 does not compromise the control of infection with Mtb strains H37Rv or 02-171. WT (circles) or TLR2−/− (squares) mice were infected intranasally with Mtb strains H37Rv or Bj 02-171 and at the indicated time points, lung cell suspensions were prepared, diluted and plated to determine the number of mycobacterial CFUs. Data points show the Mean±SEM for six mice per group. The values were found not to be significantly different as determined by the Student’s t-test. The data are representative of two independent experiments. (B) Mtb strain 02-171 presents an increased in vivo growth as compared to H37Rv or Harlingen. WT mice were infected intranasally with Mtb strains H37Rv (open circles), 02-171 (close circles) or Harlingen (triangles) and at the indicated time points, lung cell suspensions were prepared, diluted and plated to determine the number of mycobacterial CFUs. Data points show the Mean±SEM for six mice per group. The bacterial burden 24 h post-infection was Log10(CFU) 2.67±0.57, Log10(CFU) 2.45±0.76 and Log10(CFU) 2.51±0.46 (Mean±SEM for 6 (H37Rv or 02-171) or 5 (Harlingen) animals) for Mtb strains H37Rv, 02-171 or Harlingen infected mice, respectively. The statistics analysis was determined by the Student’s t-test (*,p<0.05; **p<0.01; ***,p≤0.001). (TIF) [file pone.0067277.s002.tif]
